# Quantitating myocardial fibrosis using extracellular extravascular volume determined from computed tomography myocardial perfusion imaging

**DOI:** 10.1186/s12880-024-01226-3

**Published:** 2024-02-12

**Authors:** Na Li, Xin Zhang, Jin Gu, Ming Yang, Lina Chen, Jie Yu, Heshui Shi

**Affiliations:** 1 Department of Radiology, Union Hospital, Tongji Medical College, Huazhong University of Science and Technology, 1277 Jiefang Rd, Wuhan, 430022 China; 2 Hubei Province Key Laboratory of Molecular Imaging, Wuhan, 430022 China; 3 CT Collaboration, Siemens Healthineers Ltd, Guangzhou, 510620 China

**Keywords:** Myocardial fibrosis, Extracellular volume fraction, Extracellular extravascular volume, Computed tomography, Myocardial perfusion imaging

## Abstract

**Purpose:**

Both of extracellular extravascular volume (EEV) and extracellular volume fraction (ECV) were proposed to quantify enlargement of myocardial interstitial space due to myocardium loss or fibrosis. The study aimed to investigate the feasibility of using EEV derived from myocardial computed tomography (CT) perfusion imaging (VPCT) and extracellular volume quantification with single-energy subtraction CT (ECV_− SECT_) for quantifying myocardial fibrosis.

**Methods:**

In this study, 17 patients with suspected and known coronary artery disease underwent examination using a dual-source CT scanner. The EEV_− VPCT_ was derived from dynamic whole-heart myocardial perfusion imaging, and the ECV__SECT_ was calculated from late-enhanced images 5 min after bolus contrast injection by subtracting the noncontrast baseline. The late gadolinium enhancement (LGE) on cardiac magnetic resonance (CMR) imaging was used as a reference.

**Results:**

In total, 11 patients and 73 segments exhibited positivity for LGE on CMR imaging. These were classified into three groups according to the segments: fibrotic segments (group I, *n* = 73), nonfibrotic segments in LGE-positive patients (group II, *n* = 103), and segments in LGE-negative patients (group III, *n* = 80). ECV_− SECT_, EEV_− VPCT_, myocardial blood flow (MBF), and myocardial blood volume (MBV) significantly differed among these groups (all *P* < 0.05). ECV_− SECT_ was significantly higher and EEV_− VPCT_, MBF, and MBV were significantly lower in fibrotic myocardial segments than in nonfibrotic ones (all *P* < 0.01). ECV_− SECT_ and EEV_− VPCT_ independently affected myocardial fibrosis. There was no significant correlation between ECV_− SECT_ and EEV_− VPCT_. The capability of EEV_− VPCT_ to diagnose myocardial fibrosis was equivalent to that of ECV_− SECT_ (area under the curve: 0.798 vs. 0.806, *P* = 0.844). ECV_− SECT_ of > 41.2% and EEV_− VPCT_ of < 10.3% indicated myocardial fibrosis.

**Conclusions:**

EEV_− VPCT_ is actually first-pass distribution volume that can feasibly be used to quantify myocardial fibrosis. Furthermore, the diagnostic efficacy of EEV_− VPCT_ is comparable to that of ECV_− SECT_.

## Introduction

Myocardial fibrosis is a pathological process of cardiac remodeling after myocardial infarction and is characterized by fibroblast proliferation and excessive deposition of collagen fibers in the extracellular matrix [1, 2]. Cardiac magnetic resonance (CMR) using late gadolinium enhancement (LGE) is a well-established method for the noninvasive visualization of myocardial fibrosis, and the extracellular volume fraction (ECV) derived from precontrast and postcontrast enhancement T1 mapping can be used as a quantitative metric [3, 4].

In contrast to CMR, cardiac computed tomography (CT) is a widely available, convenient, and rapid technique that has been used to quantify the enlargement of the myocardial interstitial space [5–7]. However, in this approach, ECV is determined based on the equilibrium state of contrast distribution between the myocardium and the interstitial space, which is usually reached after a long period following contrast injection (typically 5–25 min) [7–10]. To date, several imaging protocols have been proposed to calculate ECV, including single-energy CT (SECT) and dual-energy CT (DECT), and different delay time points have been used [11–14].

Recently, a few studies have reported the potential for quantifying the myocardial extracellular extravascular space using a tracer kinetic model [15, 16]. This space could be quantified using the extracellular contrast distribution volume based on the Johnson–Wilson–Lee model or the first-pass distribution volume (FDPV) based on the Tofts model. However, to the best of our knowledge, no study has investigated the feasibility of using extracellular extravascular volume (EEV) for quantitatively assessing myocardial fibrosis.

Against this background, we hypothesized that CT myocardial perfusion-derived EEV could quantify myocardial fibrosis. We aimed to: (1) assess the feasibility of using EEV in quantifying myocardial fibrosis; (2) compare EEV with ECV derived from SECT. LGE on CMR was as a reference standard.

## Materials and methods

### Study population

This study was approved by the Ethics Committee. All study participants provided written informed consent autonomously and voluntarily before their participation.

In total, 69 consecutive patients with known or suspected coronary artery disease who were prepared for coronary CT angiography (CCTA) in the clinic from March 2019 to March 2021 were prospectively recruited. Patients with contraindications for CT and CMR examinations, such as iodine contrast allergy, severe renal dysfunction (estimated glomerular filtration rate of < 30 mL/min/1.73 m^2^), or pregnancy, were excluded. All clinical examinations and laboratory tests were performed within 1 week of admission.

### Cardiac CT image acquisition

**Fig. 1:**
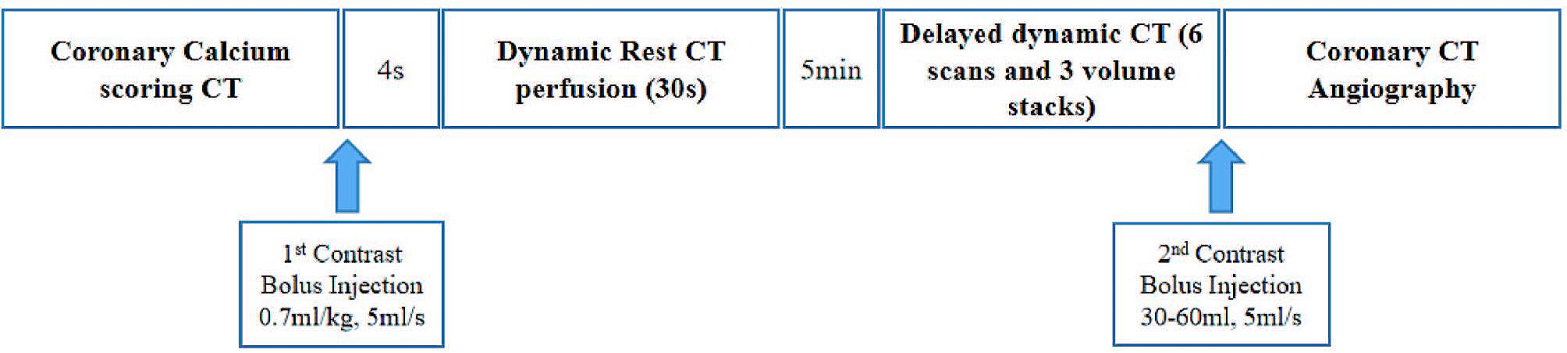
Image acquisition protocol of cardiac computed tomography

Cardiac CT was performed using a Siemens third-generation dual-source CT scanner (Somatom Force; Siemens Healthineers, Forchheim, Germany). The participants were trained to inhale and hold their breath before the examination. The total acquisition protocol is shown in Fig. 1.

The scan began with the sequence of the coronary artery calcium score. The tube voltage and tube current were automatically determined using CARE kV and CARE DOSE 4D techniques. The detector collimation was 42 × 1.2 mm, with a rotation time of 0.25 s. The slice thickness was 3 mm. Subsequently, a bolus injection of iopromide (400 mg I/mL; Bracco, Shanghai, China) was administered into the median cubital vein, followed by saline flush at an injection rate of 5 mL/s. The contrast dose was determined by weight multiplied by 0.7 mL/kg. The scan range included the regions from the tracheal bifurcation to the diaphragm.

Dynamic first pass myocardial perfusion CT (VPCT) of 30 s and delayed dynamic scan were performed during end-systole (250 ms after the R-peak) using a prospectively electrocardiogram-triggered table shuttle mode. The tube voltage was 80 kVp, and the tube current was automatically modulated (Care Dose 4D; Siemens Healthineers). The scanning parameters were as follows: detector collimation, 48 × 1.2 mm; gantry rotation time, 0.25 s/r; and slice thickness, 0.75 mm. The dynamic myocardial perfusion CT commenced 4 s before the injection of contrast medium. The delayed dynamic scan was performed 5 min after the contrast injection using exactly the same acquisition parameters as those of the myocardial first pass perfusion CT; however, only three image stacks were acquired. Finally, CCTA was performed with the following scanning parameters: detector collimation, 192 × 0.6 mm; gantry rotation time, 0.25 s/r; pitch, 0.15; and slice thickness, 0.75 mm. The tube voltage and current were automatically determined via automatic tube voltage technology (Care kV; Siemens Healthineers) and intelligent tube current scanning technology (Care Dose 4D; Siemens Healthineers). According to the body weight of the patients, 30–60 mL of iopromide (400 mg I/mL) was continuously injected into the median cubital vein, followed by saline solution, and the injection rate was 2–4 mL/s. The effective radiation dose without CCTA was 5.64 ± 1.72 mSv, which was equal to the dose–length product (DLP) multiplied by 0.014. The total DLP was 403.08 ± 123.01 mGy·cm.

### CMR image acquisition

The patients underwent CMR imaging using a 3.0 T system (Magnetom Skaya; Siemens Healthcare, Erlangen, Germany). They were repeatedly trained to inhale and hold their breath to ensure cooperation with the commands during the examination. A balanced steady-state free precession sequence and retrospective electrocardiogram gating were used to acquire left ventricular (LV) long-axis (4-, 3-, and 2-chamber) and short-axis cine images covering the entire LV layer, and 0.1 mmol/kg Gd-DTPA (Gadobutrol; Bayer AG, Berlin, Germany) was injected intravenously at rest. A T1-weighted inversion recovery gradient echo sequence was performed 10 min after the contrast injection. LGE was used for detecting myocardial scar. The scanning parameters were as follows: field of view (FOV), 380 mm × 285 mm; matrix, 216 × 256; repetition time/echo time (TR/TE), 627 ms/1.19 ms; reversal angle, 55°; and slice thickness, 8 mm. The presence of myocardial fibrosis was determined via LGE on CMR. LGE was considered if a region demonstrated a signal intensity of > 2 standard deviations (SDs) above that of a remote region within the same layer [17].

### Cardiac CT image postprocessing and analysis

Two dynamic axial image series were reconstructed using 3.0-mm-thick slices, and model-based iterative reconstruction (ADMIRE; Siemens Healthineers) was at a strength level of 3.

The early dynamic rest perfusion CT and delayed three dynamic image stacks were nonrigidly registered, and the baseline, average, and late enhancement images were generated by averaging the images before contrast arrival, images over all time points, and delayed dynamic scans (CT Dynamic Angio, *Syngo*.ViaVB 20; Siemens Healthineers). SECT CT-based ECV (ECV__SECT_) was subsequently calculated using a prototype software for research (CT Cardiac Functional Analysis Frontier, Syngo.ViaVB60 Trial version; Siemens Healthineers). Guided by average images, the software automatically contoured the left ventricle, subtracted the baseline from delayed enhancement, and then calculated ECV maps based on the following Eqs [7, 18]:

1 $$ ECV= \left(1-Hct\right)\times \left(\frac{\varDelta {HU}_{myo}}{\varDelta {HU}_{LV}}\right)$$

Where, Hct denotes the hematocrit level and ΔHU_myo_ and ΔHU_LV_ denote the pixel values in the myocardium and LV blood pool, respectively, measured from the subtracted images. The results were automatically recorded following the AHA/ACC 17-segment model.

Following nonrigid registration, the dynamic myocardial perfusion images were used for myocardial perfusion analysis (CT Myocardial Perfusion, *Syngo*.Via VB20; Siemens Healthineers). Apart from the myocardial blood flow (MBF) and myocardial blood volume (MBV), the EEV was calculated according to the Tofts model, which was reported as the first-pass distribution volume [15].

Guided by average images, MBF, MBV, and EEV maps were analyzed using a prototype software (Cardiac Function Assessment Frontier, *Syngo*.ViaVB60 Trial version; Siemens Healthineers), and the results were automatically recorded for 17 segments. The metrics measured using the two methods were evaluated separately by patient and segment according to the AHA 17-segment model, with the exception of segment 17.

### Repeatability

The measurements were performed independently by two radiologists each with 5 years of experience in cardiovascular diagnosis. The second radiologist randomly selected 11 patients to measure myocardial global and segmental ECV_-SECT_ and EEV_-VPCT_. Measurements were conducted using the same machine and software.

### Statistical analysis

Statistical analysis was performed using IBM SPSS Statistics version 26.0 (IBM Corp., Armonk, NY, USA). The normality of the data distribution for all continuous variables was assessed using the Shapiro–Wilk test. The normally distributed data were described as mean ± SD, whereas the non-normally distributed data were expressed as median (interquartile range). Categorical variables were expressed as frequency (percentage). Student’s t-test or Mann–Whitney U test was used to compare the variables between LGE-positive and LGE-negative patients, VPCT and SECT [19], respectively. One-way analysis of variance and Kruskal–Wallis test were used to compare among three groups. Furthermore, Bonferroni and Tamhanes tests were used for post-hoc comparisons between groups. C-square test or Fisher’s exact test was used to compare all categorical variables. Pearson’s and Spearman correlation coefficients were used to evaluate the correlation between continuous variables, as appropriate. Generalized estimating equation (GEE) was used to assess the effect of five metrics on myocardial fibrosis. Receiver operator characteristic (ROC) curves and corresponding area under the curve calculations were used to determine ECV/EEV thresholds derived from two methods to discriminate between normal and diseased myocardium. ROC curves were compared using DeLong test. Finally, the kappa value was used to assess the agreement of the two methods with CMR. Repeatability between observers was evaluated using intraclass correlation coefficient (ICC), and an ICC of > 0.75 was considered to indicate good interobserver agreement. *P* < 0.05 (two-tailed) was considered to indicate a statistically significant difference.

## Results

### Baseline clinical and biochemical characteristics

**Fig. 2:**
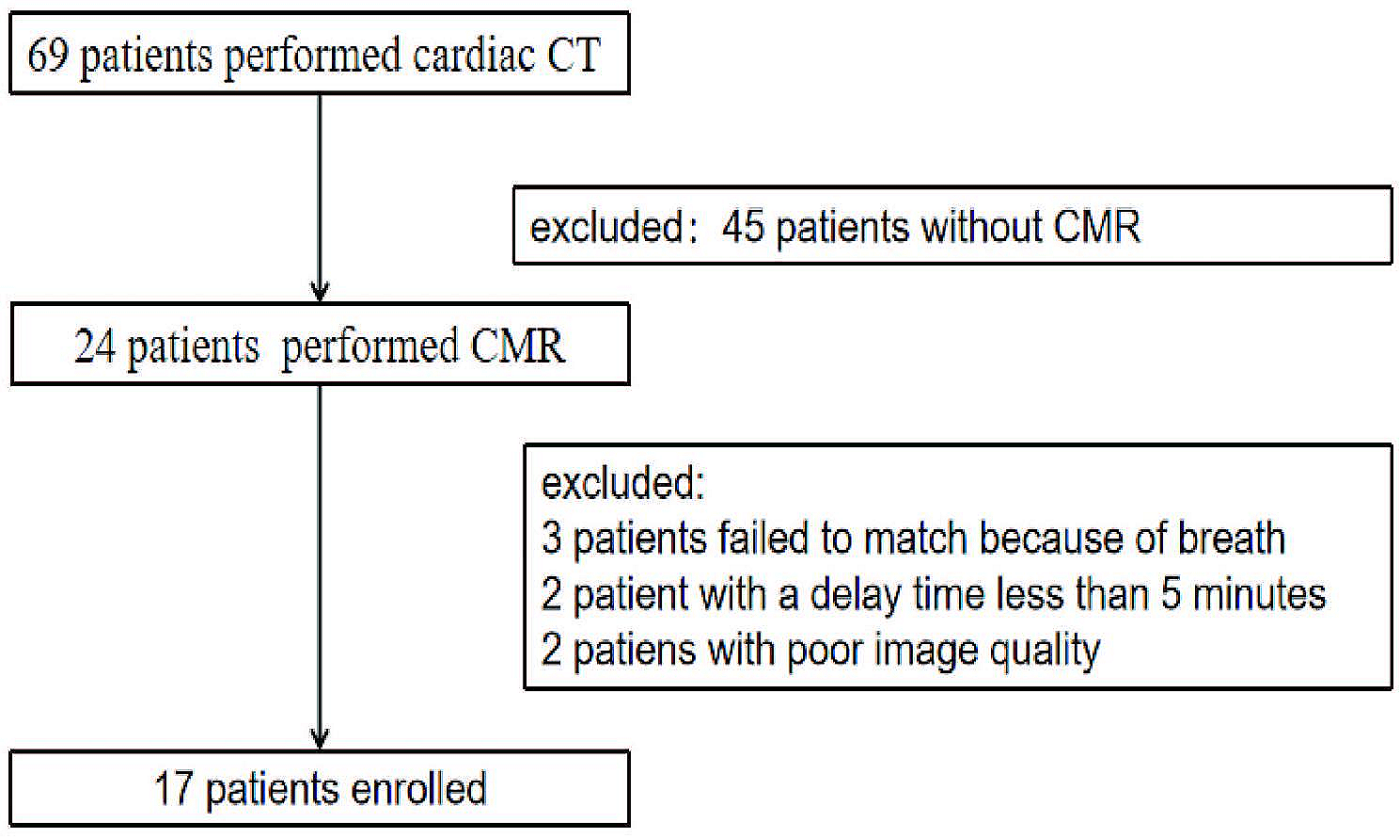
Flow chart of patients who met exclusion criteria of study

Overall, 69 patients underwent cardiac CT, excluding 45 patients who did not undergo CMR, 3 patients who experienced difficulty in respiratory motion alignment, 2 patients whose delayed enhancement did not reach 5 min, and 2 patients with poor image quality. Thus, 17 patients were included in the statistical analysis (Fig. 2). The mean age of the patients was 46.35 ± 8.92 years, and 13 (76.5%) of them were men. Of these, 7 patiens had hypertension, 4 of smoking, 4 of alcohol consumption, 1 of diabetes, 3 CRP abnormalities, 6 of Hs-cTnI abnormalities, and 5 of BNP abnormalities. CMR was used as a reference; 11 patients were positive for LGE and 6 were negative. The general clinical indicators and cardiac function parameters are shown in Table 1. No statistically significant differences were observed between the LGE-positive and -negative groups for each clinical indicator.

**Fig. 3:**
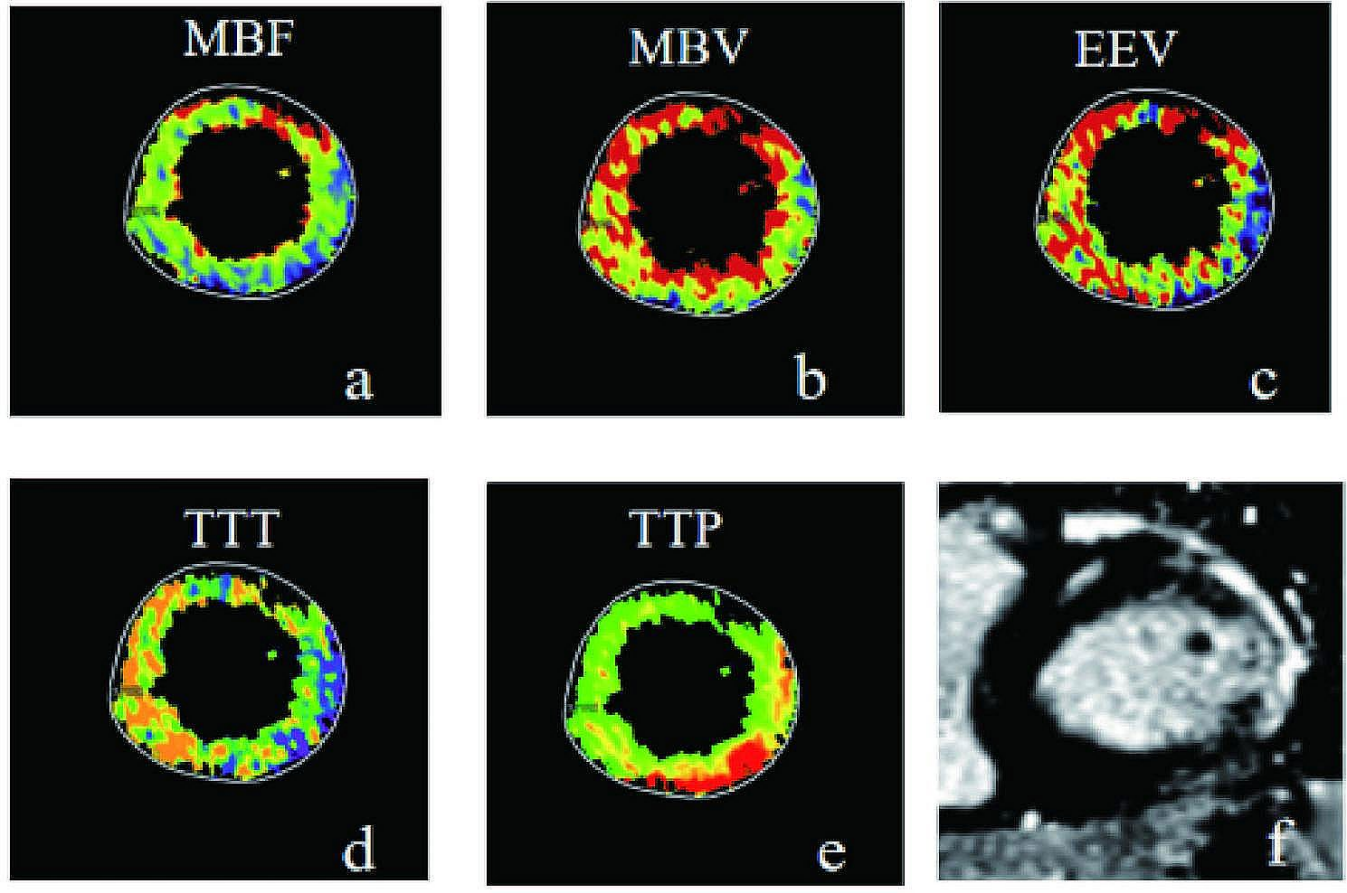
One patient had an abnormal electrocardiogram for 9 days. (**a**-**c**) Significantly decreased MBF, MBV and EEV in the lateral wall; (**d**) decreased TTT in the corresponding area; (**e**) increased TTP in the lateral wall; (**f**) CMR confirmed LGE of the lateral wall. MBF: myocardial blood flow; MBV: myocardial blood volume; EEV: extravascular extracellular volume fraction; TTT: tissue transit time; TTP: time to peak

In the analysis of myocardial segments, as 16 segments were measured incorrectly by the software owing to poor image quality, 256 myocardial segments were analyzed. These segments were classified into three groups: group I comprised fibrotic segments (*n* = 73), group II included nonfibrotic segments in the LGE-positive patients (*n* = 103), and group III included segments in the LGE-negative patients (*n* = 80). Moreover, 49 (49/73) and 57 (57/73) segments demonstrated delayed enhancement in SECT and VPCT, respectively. Figure 3 shows a patient with focal myocardial fibrosis.

**Table 1:** Demographic and clinical characteristics of the study population

Variable	Total *n* = 17		LGE positive *n* = 11	LGE negative *n* = 6	*P* value
Male (n,%)	13(76.5%)		8(72.7%)	5(83.3%)	1
Age (years)	46.35 ± 8.92		47.27 ± 7.23	44.67 ± 12.04	0.582
Height (cm)	170 ± 8.12		168 ± 5.51	173.67 ± 11.2	0.177
Weight (kg)	69.41 ± 11.71		68.18 ± 8.46	71.67 ± 16.91	0.574
BMI (kg/m2)	23.97 ± 3.18		24.19 ± 3	23.56 ± 3.77	0.71
BSA (m2)	1.89 ± 0.18		1.86 ± 0.12	1.94 ± 0.26	0.407
LVEDV (ml)	148.72 ± 46.37		156.87 ± 51.28	133.77 ± 34.74	0.342
LVESV (ml)	80.09 ± 41.58		87.96 ± 45.47	65.67 ± 31.8	0.306
SV (ml)	68.56 ± 22.57		68.82 ± 24.43	68.1 ± 20.87	0.952
LVEF (%)	48.06 ± 14.5		45.51 ± 14.34	52.75 ± 14.87	0.341
Hypertension (n,%)	7(41.2%)		6(54.5%)	1(16.7%)	0.304
Systolic pressure (mmHg)	127.76 ± 19.12		130 ± 22.69	123.67 ± 10.46	0.532
Diastolic pressure (mmHg)	82 ± 14.79		80.91 ± 16.54	84 ± 12.08	0.694
Smoker (n,%)	4(23.5%)		3(27.3%	1(16.7%)	1.000
Drinker (n,%)	4(23.5%)		2(18.2%)	1(16.7%)	0.584
Diabetes (n,%)	1(5.9%)		1(9.1%)	0	1.000
Fasting glucose (mmol/L)	4.76(4.26 ~ 5.23)		4.83(4.26 ~ 5.48)	4.81 ± 0.47	0.786
Total cholesterol(mmol/L)	4.81 ± 1.12		5.05 ± 1.28	4.36 ± 0.57	0.23
Triglyceride	1.72 ± 0.77		1.82 ± 0.83	1.54 ± 0.67	0.501
High-density lipoprotein	0.97 ± 0.25		1.02 ± 0.21	0.87 ± 0.3	0.236
Low-density lipoprotein	3.22 ± 0.92		3.38 ± 1.08	2.93 ± 0.49	0.353
HCT (%)	40.19 ± 4.4		39.43 ± 3.8	41.58 ± 5.42	0.351
CRP ≥ 8 mg/L	3(17.6%)		1(9.1%)	2(33.3%)	0.515
Hs-CRP ≥ 2.87 mg/L	3(17.6%)		1(9.1%)	2(33.3%)	0.515
CK(U/L)	81(60 ~ 143)		93.7 ± 55.45	65(61 ~ 260)	0.624
LDH(U/L)	186(170 ~ 239)		201.6 ± 36.48	171(164.5 ~ 325)	0.54
CK-MB(ng/ml)	0.85(0.38 ~ 2.92)		2.2 ± 2.03	0.5(0.3 ~ 1.98)	0.201
Hs-cTnI ≥ 26.2 ng/L	6(35.3%)		5(45.5%)	1(16.7%)	0.333
BNP ≥ 100pg/ml	5(29.4%)		5(45.5%)	0	0.102
NT-BNP ≥ 125pg/ml	1(5.9%)		0	1(16.7%)	0.353
DLP_− SECT_ (mGy*cm)	140.55 ± 43.19		151.24 ± 42.73	120.97 ± 40.09	0.175
ED_− SECT_(mSv)	1.97 ± 0.6		2.12 ± 0.6	1.69 ± 0.56	0.175
DLP_− VPCT_ (mGy*cm)	262.52 ± 80.64		281.18 ± 77.41	228.32 ± 81.58	0.206
ED_− VPCT_(mSv)	3.68 ± 1.13		3.94 ± 1.08	3.2 ± 1.14	0.206
HR (beats/min)	73 ± 11		72 ± 12	74 ± 7	0.440
CACs (AU)	0 (0 ~ 27.2)		0 (0 ~ 46.4)	0 (0 ~ 21.7)	0.713

Note: All data are expressed as the mean ± SD or medians (interquartile ranges), and number of participants (with percentages); BMI: body mass index; BSA: body surface area; LVEDV: left ventricular end-diastolic volume; LVESV: left ventricular end-systolic volume; SV: stroke volume; LVEF: left ventricular ejection fraction; LGE: late gadolinium enhancement; CK: Creatine kinase; LDH: Lactate dehydrogenase; CK-MB: Creatine kinase-MB; Hs-cTnI: highsensitivity cardiac troponin I; BNP: b-type natriuretic peptide; NT-BNP: N-terminal b-type natriuretic peptide; DLP: dose length product; ED: effective radiation dose; HR: heart rate; CACs: coronary artery calcium score

### Differences in ECV_-SECT_, EEV_-VPCT_, MBF, and MBV among the three groups

**Fig. 4:**
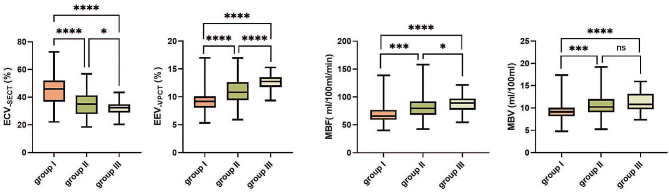
Comparison of ECV_− SECT_, EEV_− VPCT_, MBV, and MBF among the three groups

Differences in the metrics derived from the different methods are shown in Fig. 4. ECV_− SECT_ was significantly higher and EEV_− VPCT_, MBF, and MBV were significantly lower in the fibrotic myocardium than in the nonfibrotic myocardium (all *P* < 0.001). Moreover, ECV_− SECT_ in the nonfibrotic segments of the LGE-positive patients was significantly higher than that in the segments of the LGE-negative patients (*P* < 0.05). Also, EEV_− VPCT_ and MBF differed between these two groups (both *P* < 0.05).

### Difference between ECV_-SECT_ and EEV_-VPCT_ in the three groups

**Fig. 5:**
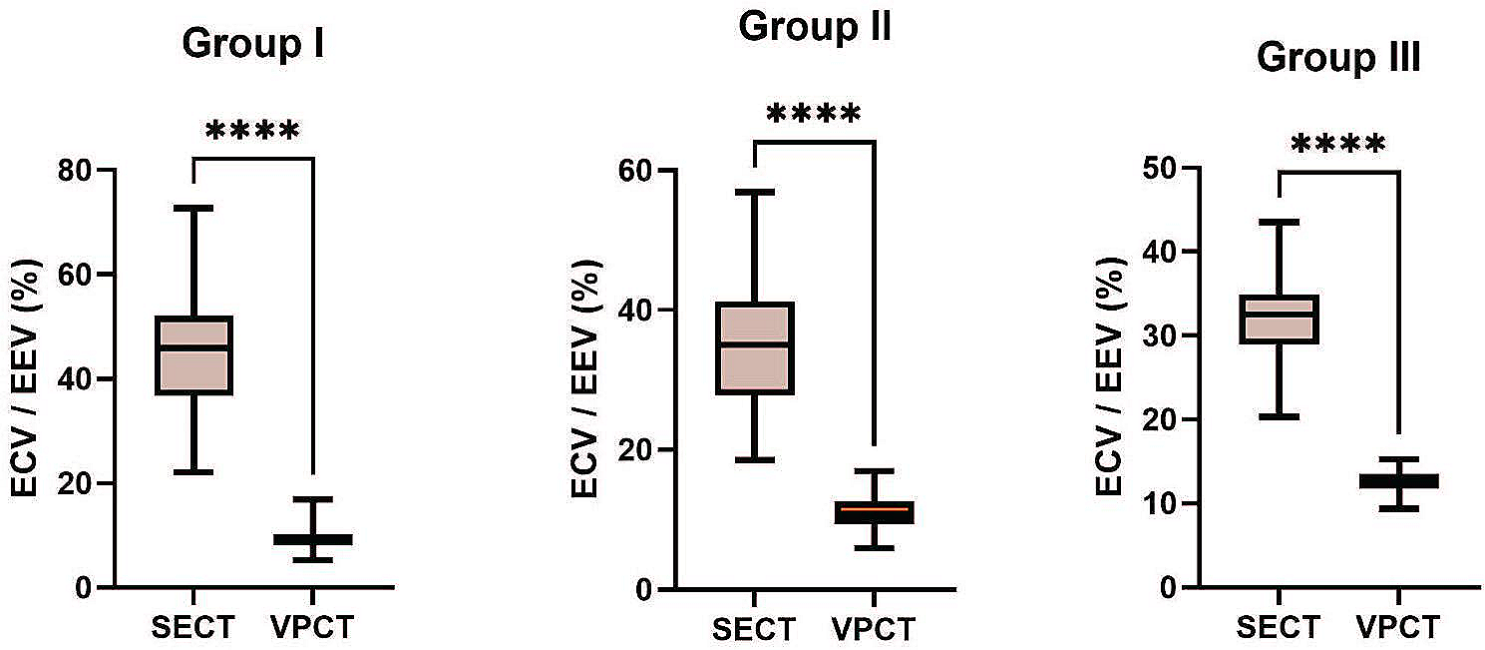
Comparison between ECV_− SECT_ and EEV_− VPCT_

Differences between the two indicators obtained using various methods are shown in Fig. 5. In the three groups, statistically significant differences were found in the values obtained using the two approaches, and EEV_− VPCT_ was significantly lower than ECV_− SECT_ (all *P* < 0.001).

### Relationship between ECV_-SECT_ and EEV_-VPCT_

**Fig. 6:**
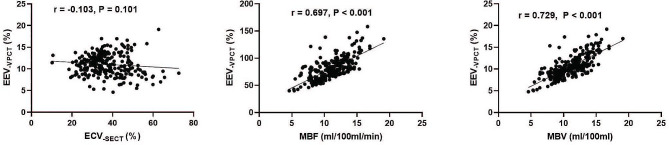
Correlations between the indicators measured via SECT and VPCT

The correlation between the indicators obtained using the two methods is shown in Fig. 6. Only a weak trend for a negative correlation between ECV_− SECT_ and EEV_− VPCT_ was observed (*r* = − 0.103, *P* = 0.101). EEV_− VPCT_ correlated significantly and positively with MBF and MBV (*r* = 0.697 and 0.729, respectively; both *P* < 0.001).

### GEE analysis for myocardial fibrosis

The influence of myocardial fibrosis is shown in Table 2. ECV_− SECT_, EEV_− VPCT_, MBF, and MBV were the factors that influenced myocardial fibrosis. ECV_− SECT_ was an independent risk factor for myocardial fibrosis, whereas EEV_− VPCT_ was a protective factor against it.

**Table 2:** The GEE analysis of myocardial fibrosis

variable	β values	wals	*P* values	OR values	95% CI
**Univariate analysis**					
ECV_− SECT_	0.137	47.915	< 0.001	1.147	1.103–1.193
EEV_− VPCT_	−0.535	42.052	< 0.001	0.586	0.498–0.688
MBF	−0.043	22.576	< 0.001	0.957	0.940–0.975
MBV	−0.325	19.357	< 0.001	0.723	0.625–0.835
**Multivariate analysis**					
ECV_− SECT_	0.146	38.651	< 0.001	1.158	1.105–1.212
EEV_− VPCT_	−0.505	18.753	< 0.001	0.603	0.480–0.758

Note: ECV and EEV are in %, MBF in ml/100 ml/min, and MBV is in ml/100 ml

### Diagnostic performance of ECV/EEV for myocardial fibrosis

The performance of ECV/EEV obtained using the two methods in diagnosing myocardial fibrosis is shown in Table 3. ECV_− SECT_ and EEV_− VPCT_ were able to diagnose myocardial fibrosis with threshold values of 41.2% and 10.3%, respectively. ECV_− SECT_ demonstrated diagnostic performance equivalent to that of EEV_− VPCT_ (area under the curve: 0.806 vs. 0.798, *P* = 0.844), whereas EEV_− VPCT_ exhibited higher sensitivity and lower specificity than ECV_− SECT_. Otherwise, the NPV of ECV_− SECT_, and EEV_− VPCT_ were about the same. When the two methods were combined, the area under the ROC curve increased to 0.821, with improved specificity and sensitivity of 84.7% and 68.5%, respectively.

**Table 3:** The diagnostic performance of myocardial fibrosis

	Cut-off value	AUC	*P* value	Sensitivity	Specificity	PPV	NPV	ACC
ECV_− SECT_	41.23	0.806	< 0.001	67.1%	83.6%	62.0%	86.4%	90.6%
EEV_− VPCT_	10.25	0.798	< 0.001	78.1%	73.2%	53.85	89.3%	74.6%

Note: ECV and EEV are in %; PPV, positive predictive value; NPV, negative predictive value; ACC, accuracy

### Repeatability and agreement test

The interobserver ICCs of ECV_− SECT_ and EEV_− VPCT_ were 0.957 (95% confidence interval: 0.943–0.968, *P* < 0.001) and 0.958 (95% confidence interval: 0.918–0.976, *P* < 0.001), respectively. The agreements of SECT and VPCT with CMR were assessed using kappa agreement coefficients, yielding values of 0.495 and 0.452 (both *P* < 0.01), respectively.

## Discussion

Myocardial ECV provides information on the distribution of cells (primarily represented by myocyte masses) and interstitium (extracellular matrix and intravascular space), which reflect the extent of myocardial fibrosis [3]. This study investigated the correlation between ECV_-SECT_ and EEV_-VPCT_ and evaluated their performance in diagnosing myocardial fibrosis.

SECT- and DECT-based ECV have been employed to assess the extracellular interstitium in cardiovascular diseases [20–22]. However, to the best of our knowledge, the comparison of VPCT-based EEV and SECT-based ECV has not been reported. The EEV-VPCT, MBF, and MBV of injured myocardium were significantly lower than those of normal myocardium. In addition, the ECV of the nonfibrotic segments in the LGE-positive group was higher than that of the segments in the LGE-negative group, whereas EEV, MBF, and MBV exhibited the opposite trend. This finding indicates that the nonfibrotic myocardium distal to the fibrotic myocardium may exhibit subtle abnormalities and is in a suboptimal state. After myocardial infarction, fibrosis extends over time to noninfarcted areas. Reactive myocardial fibrosis occurs in the peri-infarcted and distal segments of the infarcted tissue, thereby affecting cardiac systolic and diastolic function [23]. This distal myocardial fibrosis is of great importance and will lead to a reduction in cardiac output and deterioration in cardiac function, increasing the likelihood of heart failure and the risk of death in the long term [24]. Our study showed that VPCT allowed to monitor hemodynamic abnormalities in distal segments of scarred myocardium and assess peri-infarct viable myocardium so that early action can be taken to prevent myocardial scar enlargement in patients.

Interestingly, we found that EEV decreased in the fibrotic myocardium, which is inconsistent with the findings described in some previous reports. Pack [25] and So et al. [16] reported that EEV was elevated in the infarcted myocardium compared with the level in the normal myocardium. The loss of viable myocardial cells and the high permeability of the cell membrane of necrotic cells might have increased EEV. In the three-compartment model (intravascular, interstitial, and intracellular), ECV includes EEV and vessel volume, and assuming that vessel volume is constant, EEV and ECV should theoretically be positively correlated or exhibit the same trend as ECV. However, a study revealed comparable findings to our study, reporting the opposite of what is expected, i.e., a decrease in EEV in the infarct area [26]. The decrease in EEV reported in the previous study might have been due to the shorter duration of dynamic-enhanced perfusion scans or the absence of recurrent flow. Conversely, in this study, EEV decreased in the injured myocardium because it did not signify what is commonly considered as the EEV. The EEV derived from the first pass CT perfusion was preliminary proposed by Mahnken [15] et al. It was initially a parameter called FPDV and was thought to reflect the myocardial interstitial space. In contrast, ECV usually derived from Delayed-enhanced CT or MRI at the equilibrium state. Mahnken et al. reported a significant decrease in the FPDV in the ischemic myocardium [15]. The EEV obtained by us is consistent with this result. The FPDV of the fibrotic myocardium is significantly smaller than that of the nonfibrotic myocardium, with an FPDV of < 10.25 mL/100 mL, suggesting myocardial fibrosis. Therefore, a significant difference was noted between EEV_-VPCT_ and ECV_-SECT_, because these were two different metrics that represent different organizational characteristics. Ugander [27] et al. performed cardiac magnetic resonance ECV imaging of 126 patients and found a mean ECV of 51% ± 8% for infarcted myocardium. The threshold of our ECV_-SECT_ was close to its lower limit.

Ohta et al. reported a good correlation between ECV derived from subtraction CT and iodine concentration [6]. Conversely, the subtraction in this study was based on dynamic perfusion imaging and not on delayed iodine-enhanced CT. The results of the study showed a significant positive correlation of EEV with MBF and MBV. Based on the fact that EEV is actually FPDV, this is expected. CT perfusion quantification parameters were modeled using a deconvolution technique for the time attenuation curve (TAC) and arterial input function (AIF) curves: MBF = MaxSlope (Tissue TAC)/Maximum (AIF), FPDV = MaxEnhancement (Tissue TAC)/Maximum (AIF) [15]. The FPDV of iodine contrast agent in human tissue is related to tissue blood flow. Scarred myocardium exhibits decreased perfusion, decreased FPDV.

There were no differences between the clinical characteristics of LGE-positive and LGE-negative patients. This may be due to the fact that our subjects were patients with chronic myocardial infarction (MI), so the laboratory indices did not show significant abnormalities. In addition, the small sample size may also be a factor. This precisely demonstrated the value of our analysis from the segment level. Obviously, it was important to quantify myocardial viability at the level of the myocardium and assess myocardial hemodynamic changes in MI patients.

This study preliminarily explored the diagnostic performance of EEV_-VPCT_ for focal myocardial fibrosis. The previous study suggested that CT myocardial perfusion may be a more reliable predictor of viability than extravascular contrast distribution volume in the setting of severe microvascular obstruction with severely reduced myocardial blood flow [16]. As only first-pass dynamic scan was needed and contrast-to-noise ratio was usually relatively higher than the delayed phase, EEV could be a reliable metric for evaluation of enlargement of myocardial interstitial space, such as myocardial fibrosis or scarring, within shorter exam time. Combined EEV_-VPCT_ and ECV_-SECT_ can be used to quantitatively evaluate myocardial viability in MI patients and to assess myocardium at risk (MAR). The information can guide the revascularization. It is also essential for assessing the therapy efficacy in MI patients and for monitoring hemodynamic recovery. In the future, it will be useful to assess hemodynamic and microcirculatory changes in non-ischemic cardiomyopathy, as well as myocardial viability and therapeutic efficacy in MI.

This study has certain limitations. These results are based on a small number of patients recruited from a single center over a period of 2 years. Given the complexity of the study and factors such as investigator availability and competing studies, further hindered by a COVID-19 outbreak during 2019 to 2021, patient recruitment was significantly restricted. Second, the radiation dose is a non-negligible concern. However, we reduced the radiation dose using techniques such as automatic tube voltage regulation and iterative reconstruction. Finally, we did not evaluate the correlation between CMR-derived ECV and ECV_-SECT_/EEV_-VPCT_. The patients did not undergo T1 mapping image acquisition for quantitative ECV assessment. The value of ECV quantification is in detecting diffuse myocardial fibrosis not revealed by LGE, and all patients in our study showed focal myocardial fibrosis.

## Conclusions

In conclusion, this study demonstrate a non-significant association between ECV_− SECT_ and EEV_− VPCT_. EEV based on myocardial perfusion CT is actually FPDV, which can identify myocardial focal fibrosis. Moreover, the diagnostic performance of ECV_− VPCT_ is equivalent to that of ECV_− SECT_.

## Data Availability

The data presented in this study are available on reasonable request from the corresponding author.

## Abbreviations

CCTA: Coronary CT angiography
CMR: Cardiac magnetic resonance
CT: Computed tomography
CTP: Computed tomography perfusion
DECT: Dual-energy CT
DLP: Dose–length product
ECV: Extracellular volume fraction
EEV: Extracellular extravascular volume
FDPV: First-pass distribution volume
ICC: Intraclass correlation coefficient
LGE: Late gadolinium enhancement
MBF: Myocardial blood flow
MBV: Myocardial blood volume
ROC: Receiver operator characteristic
SECT: Single-energy CT
VPCT: Myocardial perfusion CT
